# Vision Is Adapted to the Natural Level of Blur Present in the Retinal Image

**DOI:** 10.1371/journal.pone.0027031

**Published:** 2011-11-02

**Authors:** Lucie Sawides, Pablo de Gracia, Carlos Dorronsoro, Michael A. Webster, Susana Marcos

**Affiliations:** 1 Instituto de Óptica, Consejo Superior de Investigaciones Científicas (CSIC), Madrid, Spain; 2 Department of Psychology, University of Nevada, Reno, Nevada, United States of America; University of Antwerp, Belgium

## Abstract

**Background:**

The image formed by the eye's optics is inherently blurred by aberrations specific to an individual's eyes. We examined how visual coding is adapted to the optical quality of the eye.

**Methods and Findings:**

We assessed the relationship between perceived blur and the retinal image blur resulting from high order aberrations in an individual's optics. Observers judged perceptual blur in a psychophysical two-alternative forced choice paradigm, on stimuli viewed through perfectly corrected optics (using a deformable mirror to compensate for the individual's aberrations). Realistic blur of different amounts and forms was computer simulated using real aberrations from a population. The blur levels perceived as best focused were close to the levels predicted by an individual's high order aberrations over a wide range of blur magnitudes, and were systematically biased when observers were instead adapted to the blur reproduced from a different observer's eye.

**Conclusions:**

Our results provide strong evidence that spatial vision is calibrated for the specific blur levels present in each individual's retinal image and that this adaptation at least partly reflects how spatial sensitivity is normalized in the neural coding of blur.

## Introduction

Optical aberrations degrade the quality of the images projected onto the retina and vary widely in magnitude and distribution across the population [1]. Unlike spherical or cylindrical errors, High Order Aberrations (HOAs) are not typically corrected, and thus individuals are each chronically exposed to different patterns of retinal blur. We asked whether spatial coding in the visual system is matched to the native blur level specific to an individual's HOAs. Recent studies have demonstrated short-term aftereffects in both perceived blur and visual acuity following exposure to blur introduced optically or by filtering images [2], [3]. Several studies show evidence that eyes are adapted to HOA induced by corneal pathology [4], by corneal surgery [5] or by aging [6]. We have shown that observers can adapt to the blur induced by HOA from scaled versions of their own aberrations, or those from other subjects [7]. However, the extent to which observers are adapted to their own optical aberrations remains unresolved. On the one hand, both short- and long-term adaptation can selectively adjust to the axis of astigmatic blur [8], [9], and visual performance is better in observers with optics strongly degraded by corneal pathology compared to normal subjects induced with similar amounts of HOAs [4]. Moreover, Artal et al. [10] found that stimuli seen through an individual's natural aberrations appear sharper than when seen through a rotated version of the same aberrations, and adaptation to surgically induced HOAs has been suggested to occur in patients after LASIK surgery [5]. These studies thus point to neural compensations for the wave aberrations characterizing the individual's eye. Yet on the other hand, studies of visual acuity and subjective image quality have found immediate improvements after correcting HOAs, with only a small residual bias toward the observer's native HOAs and little further improvement with training [11], [12]. These results have therefore suggested that there may instead be relatively little adaptation to HOAs. To directly test for this adaptation, we used a custom-developed Adaptive-Optics (AO) system to measure and correct the observer's aberrations (with best spherical refraction error correction and a 5-mm pupil). By removing the natural aberrations of the eye, all observers were exposed to identical aberration patterns and therefore any difference in the visual response must be due to neural factors. We then manipulated retinal blur by projecting degraded images with known HOAs. Subjective focus was measured with a 2-Alternative-Forced-Choice (2AFC) procedure in which the observer had to report whether the image displayed on the monitor appeared “too blurred” or “too sharp”.

## Results

### Testing scaled HOA patterns

**Figure 1:**
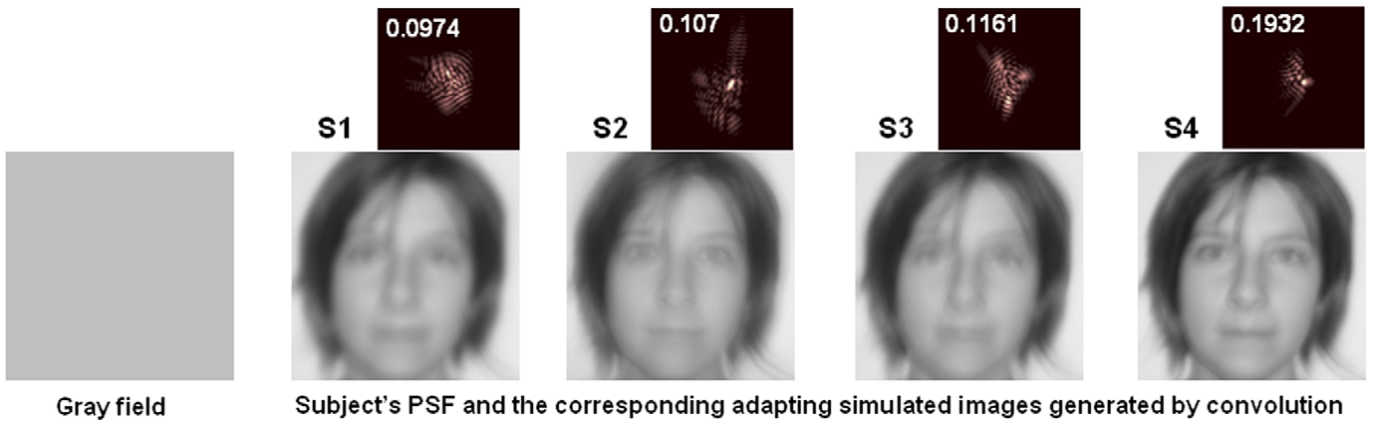
Testing scaled high order aberrations patterns. a) Adapting images in testing scaled high order aberrations patterns: Gray field and simulated adapting images generated by convolution with the PSFs (shown, with corresponding SR) obtained from 4 different subjects' HOA patterns. Tilt and astigmatism were set to zero whereas defocus was adjusted to maximize optical quality. Data are for 5-mm pupils.

**Figure 2:**
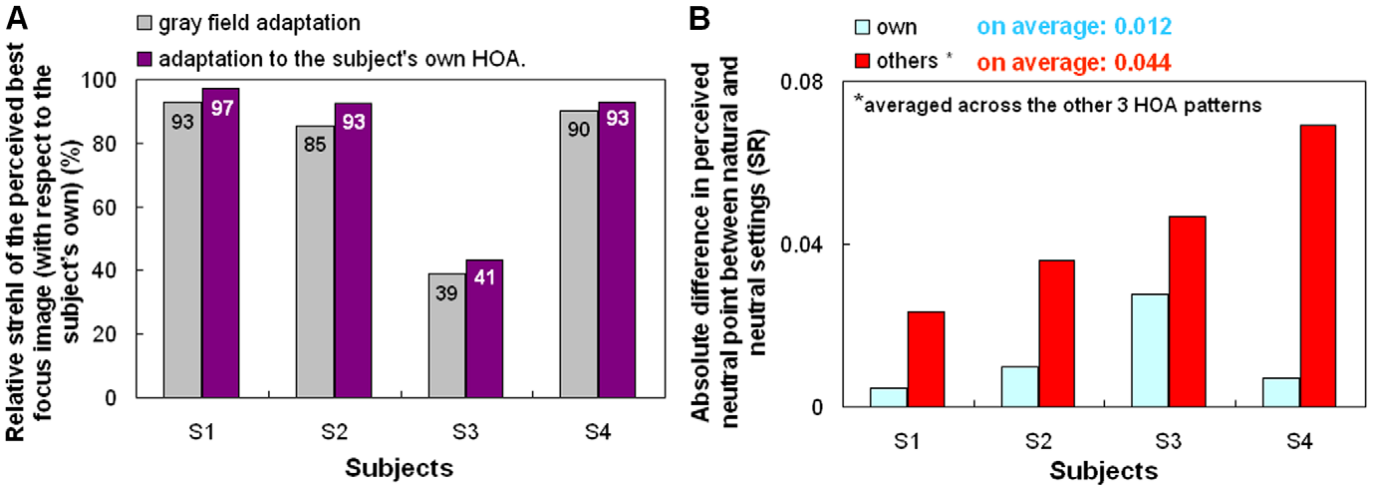
Testing scaled high order aberrations patterns. a) Relative Strehl Ratio of the perceived best focus image (with respect to the subject's native level) for gray field adaptation or adaptation to each subject's own HOAs. b) Difference in Strehl Ratio between gray and natural adaptation when subjects were adapted to their own HOAs (blue) and other subjects' HOAs (red), averaged across the other 3 HOA patterns.

**Figure 3:**
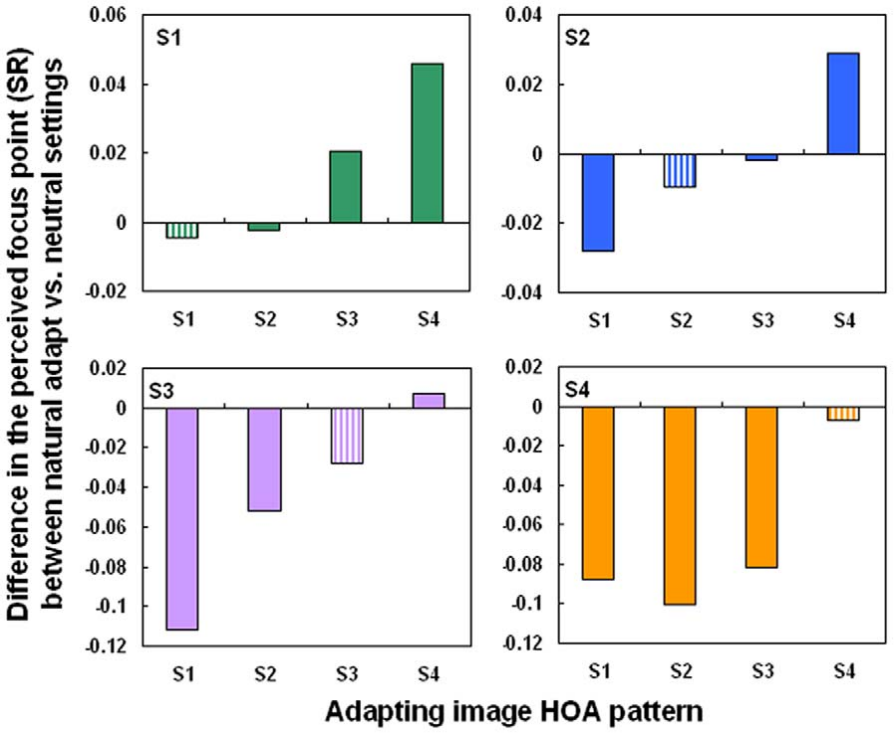
Testing scaled high order aberrations patterns. Difference in the perceived focus level (in terms of Strehl Ratio) between natural adaptation and the subject's neutral settings, when the subjects adapted to their own aberrations (striped bars) or to the aberrations for each remaining subject (solid bars).

In the first experiment, sequences of images were blurred by convolution with the corresponding point spread functions (PSFs) estimated from scaled versions of each observer's HOA patterns, ranging from diffraction limited (scale factor F = 0) to double the amount of natural blur (F = 2) in 0.05 steps. When F = 1, the simulated image thus represents the natural degradation imposed by the subject's HOAs (Fig 1). The blur level selected as best focused was very close to the natural blur level for 3 of 4 observers, and for all observers remained very similar whether observers first adapted to a neutral gray field or to the image filtered by their own natural blur. The settings under neutral adaptation (gray field) were roughly 77% of their HOA (89.3% excluding Subject S3 who has a low tolerance to blur) and similar to the settings when they were adapted to their own HOA (82%, and 94.3% excluding S3) (Fig 2A). The neutral focus therefore was not perceived for fully corrected optics, and in general, occurred at a blur level near the subject's own aberrations. The low difference in the perceived neutral focus between neutral adaptation (gray field) and natural adaptation suggested that the subjects were “pre-adapted” to their own aberration level. The average difference in the perceived focus between the neutral and the natural adaptation conditions was 0.012 (in terms of strehl ratio, SR, defined as the normalized peak of the PSF). However, the perceived focus was biased from the blur level predicted by their HOAs (by 0.044 in terms of SR, on average) when each observer was instead adapted to images blurred by other's HOA patterns, corresponding to the native blur of the other 3 participants (Fig 2B). The pattern of after-effects was consistent with the adaptation predicted by the overall blur magnitude. Specifically, in individuals with lower SRs (more native blur), adaptation to the blur from the less aberrated eyes caused their native focus level to appear too blurred (as expected if they were now adapted to images that were previously for them “too sharp” Fig 3, observer S1). Conversely, observers with low levels of natural blur perceived their natural focus level as too sharp when adapted to the blur from more aberrated eyes (Fig 3; observer S4).

### Testing 128 real complex HOA patterns

**Figure 4.Testing:**
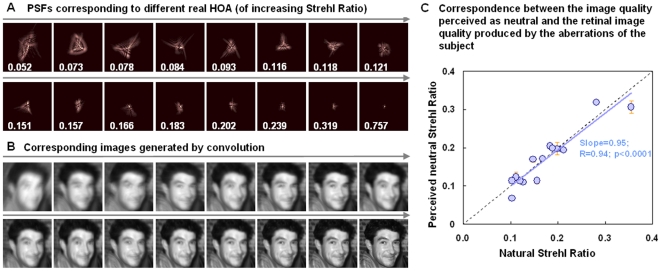
Testing 128 real complex high order aberration patterns. a) Subset of 16 PSFs estimated from HOA in real eyes (from a total of 128 used in the blur judgment experiment), with their corresponding SR. Tilt and astigmatism were set to zero whereas defocus was adjusted to maximize optical quality. Optical quality ranges from highly degraded from surgical eyes to almost diffraction-limited (from AO-correction). Data are for 5-mm pupil diameters. b) Test sequence images blurred by convolution with the corresponding PSFs in a). The experiment used the complete sequence of 128 images. c) Strehl Ratio of the image perceived as best focused versus the natural Strehl Ratio for each of the 15 subjects.

In a further experiment, we examined whether the internal norm for blur is set to a specific aberration pattern or to the overall blur, regardless of its form. Observers again judged whether images appeared too blurred or too sharp, but this time for an image sequence generated from a set of 128 different HOA patterns from real eyes. These ranged from very pronounced HOAs (from surgically corrected eyes) to almost diffraction-limited (achieved with AO-correction measurements), with blur levels (SR) ranging from 0.049 to 0.757 (5-mm pupils). A subset of PSFs and the corresponding simulated images generated by convolution are shown in Fig. 4 (A, B). Fifteen subjects were tested, with SR ranging from 0.103 to 0.356 (5-mm pupils). There was a close correspondence between the image quality perceived as neutral and the retinal image quality produced by the aberrations of the subject, with an average deviation of 0.014 (in terms of SR), and a strong correlation between the blur of the image perceived as neutral and the subject's own blur (Slope = 0.95; R = 0.94; p<0.0001; Fig. 4C). For the majority of the subjects, the blur level perceived as best focused was well predicted from the magnitude of the native blur present in their eyes.

## Discussion

Adaptive Optics (AO) is a useful technique to compensate the aberrations of the subjects, as had been shown in numerous previous studies [13]–[15]. AO, allowing to appropriately control the blur level of the retinal image, provided a powerful technique to directly test neural adaptation to the subjects' own blur level. The innovative finding of the paper is that subjects appear to be adapted to the blur level imposed by their own optical aberrations. Adaptive Optics has allowed us to cancel the natural aberrations of all subjects, exposing observers to identical aberration patterns and ensuring that any difference across subjects will arise from their own neural processing and their prior neural adaptation. Under these conditions, we found that an observer's focus settings remain largely unaffected when adapting to their own aberrations, but were significantly biased toward higher or lower blur levels when adapted to the aberrations from observers with more or less optical blur respectively. This demonstrates that the visual mechanisms mediating the perception of focus can differentially adapt to changes in image blur level resulting from HOAs. Moreover, the finding that aftereffects were weakest near the level of the observer's natural blur (Fig 4, S1 and S4) further suggests that the individual's subjective neutral point corresponded to the long-term adapted state induced by their optics. This in turn suggests that the blur level that appears correctly focused to an observer is not merely a learned criterion (e.g. so that all observers encode blur similarly but choose the blur level they are accustomed to seeing). Specifically, if observers differed only in how they labeled the blur (and thus did not differ in the neural encoding) then they should all show the same aftereffects for a given adapting level, regardless of whether they described that level as too blurred or too sharp (since the adaptation would induce the same shifts in their neural sensitivity). Instead, direction of the blur aftereffect was specific to each observer's intrinsic blur level, revealing that the individual differences in perceived focus at least in part reflect differences in how their sensitivities are normalized to their ambient blur level [16].

Our results also show that the close association between the coded norm for blur and the observer's aberrations holds over a very wide range of native blur levels (Fig 3). For the majority of subjects, the blur level that is perceived as best focused is very closely predicted from the magnitude of the native blur present in their eyes. Together with the observed adaptation effects, this finding strongly suggests that the perception of focus is calibrated for the specific blur levels present in each individual's retinal image. On the other hand, this normalization may depend largely on the overall level of the blur and not on the specific pattern of HOAs generating this level, for this close association held even though the stimuli were not matched to the observers in terms of the actual form of the HOAs. This raises the possibility that the processes of blur adaptation may be unable to resolve subtle differences in the patterns of blur specific to different HOAs, so that the adaptation state is instead largely controlled only by the blur magnitude. The fact that the overall amount of blur proved more critical than orientation is a further novel finding of the study. However, this does not preclude the possibility that the adaptation can also selectively adjust for some differences in the HOA pattern when blur magnitudes are equated [10] analogous to the selectivity found for low order aberrations [8].

How can these results be reconciled with evidence for only weak adaptation to HOAs? A likely answer is that different studies have measured different perceptual judgments. Correcting HOAs leads to improvements in visual acuity and an increased subjective impression of sharpness [12], [15], [17]. Previous studies testing for adaptation after correction selected the sharpest image for best image quality, while our observers were instead required to choose the point of subjective focus at which the image appeared neither blurred nor sharp. Consistent with this difference, we scaled the PSF by factors ranging from 0 to 2, which ranged from sharper to blurrier than their natural HOA, whereas previous studies (e.g.[11]) instead used stimuli ranging from -1 to 1, and thus never increased the blur relative to the natural level. It is thus plausible that the much stronger implied adaptation we observed is because this adaptation is more conspicuous in how it affects judgments of perceived focus, which may correspond to the neural norm for blur perception [18]. This norm is set by the observer's natural level of blur, yet as we have shown can be rapidly recalibrated when adapted to a different level of HOAs. Consequently spatial vision may be normalized to compensate for the optical imperfections of the eye in the same way that color vision is normalized to discount the spectral filtering of the lens [19].

## Materials and Methods

### Ethics Statement

All participants, who were acquainted with the nature of the study, provided written informed consent. All protocols met the tenets of the Declaration of Helsinki and had been previously approved by the Consejo Superior de Investigaciones Científicas (CSIC) Ethical Committee.

### Subjects

Four experienced observers participated in the first experiment and 15 observers (3 of the authors and 12 naive observers) participated in experiment 2. All had normal vision, their natural Strehl Ratio at best focus varied from 0.097 to 0.356 (0.097 to 0.1932 in experiment 1 and 0.103 to 0.356 in experiment 2).

### Apparatus and Stimuli

The primary components of our custom Adaptive Optics system are a Hartmann-Shack wavefront sensor (HASO 32 OEM, Imagine Eyes, France) and an electromagnetic deformable mirror (MIRAO, Imagine Eyes, France). A motorized Badal system compensated for the subject's spherical error and two psychophysical channels were used for stimulus presentation. The first channel, composed of a 12 mm×9 mm SVGA OLED minidisplay (LiteEye 400) ,was used to fix the sight during the measurement and correct the subject's aberration; The second, composed of a 12×16 inches CRT Monitor and controlled by the ViSaGe psychophysical platform (Cambridge Research System, UK), was used to project the adapt and test images. The system was controlled using custom routines written in Visual C++ (to control the AO-loop and the Badal system) and Matlab to control the ViSaGe psychophysical platform. More details of the AO-system are reported in recent studies [15], [17].

Psychophysical measurements were performed under static correction of aberrations. We performed a close loop correction at 13 Hz in 15 iterations and saved the state of the deformable mirror with the voltage applied to each actuator for future use. The residual wavefront error was continuously monitored (before and after each measurement) and deemed satisfactory if less than 0.15 µm RMS (excluding tilts and defocus). On average, RMS (excluding tilts and defocus) decreased from 0.523±0.33 µm to 0.080±0.038 µm, with an average HOAs correction of 82.0±8.5%.

### Generation of the optical blur

The original image (a face) in both experiments was acquired using a photographic digital camera with an original resolution of 4 M pixels and converted to grayscale. In the first experiment, testing scaled HOA patterns, the optical blur was generated by convolution of the image with the PSF estimated from the subject's HOAs. Aberrations of the subject were measured using the AO-set-up, and fitted by 7^th^ order Zernike polynomials. Tilts and astigmatism were set to zero whereas defocus was set to optimize SR and achieve best optical quality. Standard Fourier Optics techniques [20], were used to calculate the corresponding Point Spread Function PSF. The PSF was scaled to match the pixel-size of the face image in 1.98° window. All computations were performed for 5-mm pupils. The Stiles-Crawford effect was not considered, as for a typical values (<0.1 mm^−1^) [21] its effect was negligible for the purposes of our study. A double diffraction when viewing the convolved image through a diffraction-limited 5-mm pupil (convolution + artificial pupil-aperture) was not corrected by means of inverse filtering as also considered negligible. Simulations conducted to assess the impact of these two effects revealed that the effect on the final contrast of convolved E targets with similar levels of blur to those used on the experiment was less than 10% with respect to the contrast obtained without including these two factors. Simulations and experiments using a CCD camera as an artificial retina confirmed that the convolved images were optically corrected both in scale and contrast, within the experimental error of the CCD image acquisition. Sequences of images were generated for each subjects' HOAs, by multiplying each Zernike coefficient by a factor F between 0 and 2 in 0.05 steps. Multiplying the Zernike coefficients by these factors modifies the amount of blur while preserving the relative shape of the PSF. Each set of testing images contained 41 different test images ranging from diffraction-limited to double the amount of natural blur. When the factor F was equal to 1, the simulated image represented the natural degradation imposed by the subject's HOAs and these images were used in the conditions testing adaptation to natural aberrations.

In the second experiment, testing 128 real complex HOA patterns, the optical blurred was generated by convolution, using the same method as in first experiment, with the PSF estimated from 128 different complex aberration patterns from real eyes. Tilts and astigmatism were set to zero, whereas defocus was set to optimize Strehl Ratio and achieve best optical quality. The optical quality ranged from high amounts of HOAs (from surgically altered eyes) to almost diffraction-limited (achieved with AO-correction measurements). The sequence of test images thus contains 128 images with Strehl Ratio ranging from 0.049 to 0.757 (5-mm pupils).

### Procedure

Observers viewed the images in a darkened room. An artificial pupil in a pupil conjugate plane guaranteed that the measurements were performed under constant pupil size of 5-mm pupil diameter. The subject's pupil was aligned to the system using a bite bar and the pupil was centered and focused. The subject was then asked to adjust the best subjective focus, by controlling the Badal system with a keyboard while he/she looked at a high contrast Maltese cross on the minidisplay.

Natural aberrations were measured and corrected (all aberrations except tilts and defocus) in a closed loop adaptive optics operation. Then, the subject was asked again to adjust the Badal system position that provided the sharpest subjective focus for this AO-corrected condition. The state of the mirror that achieved this correction was saved and applied during the measurements. During testing, the natural pupil was continuously monitored to ensure centration, and the wave aberration was measured before and after each test (i.e. every 5 minutes) to ensure appropriate AO-correction (with a new closed-loop correction applied if necessary).

The images in the tests were presented on the CRT monitor and subtended 1.98 degrees. The psychophysical paradigm consisted of a 2AFC procedure, where the subject responded whether the image was sharp or blurred. Stimulus levels were varied with a quest algorithm in order to find the level of best perceived focus point for a given adaptation condition (neutral adaptation with a gray-field or adaptation to natural aberrations). In all cases, the sequence of the psychophysical experiment consisted of 1 min exposure to the adapting image after which a test image was presented to the subject who had to respond if the image was sharp or blurred. The subject re-adapted for 3 seconds between each test image. Adapting images were spatially jittered in time to prevent local light adaptation.

In the first experiment, blur judgments were measured on 4 subjects (Strehl Ratio ranging from 0.094 to 0.1932 (5-mm pupils), after neutral adaptation (gray-field) and after adaptation to images blurred with the natural degradation imposed by each of the 4 subject's HOAs (including their own). The results were analyzed in terms of the SR of the perceived focus point. In a second experiment, judgments of perceived blur were measured in 15 subjects to determine for each individual the physical blur level that appeared best focused under neutral adaptation (gray field). Typically 3 repeated settings were made for each observer.
